# The Genopolis Microarray Database

**DOI:** 10.1186/1471-2105-8-S1-S21

**Published:** 2007-03-08

**Authors:** Andrea Splendiani, Marco Brandizi, Gael Even, Ottavio Beretta, Norman Pavelka, Mattia Pelizzola, Manuel Mayhaus, Maria Foti, Giancarlo Mauri, Paola Ricciardi-Castagnoli

**Affiliations:** 1 Department Informatics, Systemistics and Communication, University of Milano-Bicocca, Via degli Arcimboldi 8, Milano, Italy; 2 Department of Biotechnology and Bioscience, University of Milano-Bicocca, Piazza della Scienza 4, Milano, Italy

## Abstract

**Background:**

Gene expression databases are key resources for microarray data management and analysis and the importance of a proper annotation of their content is well understood.

Public repositories as well as microarray database systems that can be implemented by single laboratories exist. However, there is not yet a tool that can easily support a collaborative environment where different users with different rights of access to data can interact to define a common highly coherent content. The scope of the Genopolis database is to provide a resource that allows different groups performing microarray experiments related to a common subject to create a common coherent knowledge base and to analyse it. The Genopolis database has been implemented as a dedicated system for the scientific community studying dendritic and macrophage cells functions and host-parasite interactions.

**Results:**

The Genopolis Database system allows the community to build an object based MIAME compliant annotation of their experiments and to store images, raw and processed data from the Affymetrix GeneChip^® ^platform. It supports dynamical definition of controlled vocabularies and provides automated and supervised steps to control the coherence of data and annotations.  It allows a precise control of the visibility of the database content to different sub groups in the community and facilitates exports of its content to public repositories.  It provides an interactive users interface for data analysis: this allows users to visualize data matrices based on functional lists and sample characterization, and to navigate to other data matrices defined by similarity of expression values as well as functional characterizations of genes involved. A collaborative environment is also provided for the definition and sharing of functional annotation by users.

**Conclusion:**

The Genopolis Database supports a community in building a common coherent knowledge base and analyse it. This fills a gap between a local database and a public repository, where the development of a common coherent annotation is important.  In its current implementation, it provides a uniform coherently annotated dataset on dendritic cells and macrophage differentiation.

## Background

### The Genopolis gene expression database

The Genopolis Consortium operates an Affymetrix Genechip^® ^service, specialized in the transcriptional profile of cells and tissues related to the immune system and to the area of immunopathology.

Large-scale gene expression analysis is of great relevance in the field of immunology to generate a global view of how the immune system attacks invading micro-organisms, maintains tolerance, creates a memory for past infections: fundamentals questions in immunology address how the immune system distinguish between self and non-self, and how immune cell differentiation and growth is regulated.

The Genopolis Microarray Database was designed as a resource to support a focused scientific community and it was deployed to support the community studying dendritic cells functions and host-parasite interactions.  We present here both the software system and its current implementation.  The system presents a selection of features that differs from other microarray databases and that is ideal to support distinct groups of users working on a common subject.  In its current implementation, it provides gene expression data on a precise biological system that are homogeneous in terms of the measurement platform and the annotation process used.

### Annotation of microarray data

The importance of the characterization of microarray experiments is well understood [1]: a proper description of experiments' conditions and processes is a necessary condition to evaluate data generated with different experiment designs and instrumentations.

A set of guidelines called MIAME (Minimum Information About a Microarray Experiment) [2] was proposed by the Microarray Gene Expression Data Society [3].  MIAME is a document that lists a minimum set of information to characterize a microarray data set. This includes information about the experiment design, the targeted experimental factors, the organism studied, the measurement platform and all the biological or data processing protocols that have been applied in order to extract data from the biological material.

A further effort has been made to define a representation of this information that can be machine-processable and may be used for data exchange across microarray related software applications.  Result of this effort is the Micro Array Gene Expression Object Model (MAGE-OM), and its corresponding XML data exchange format, MAGE-ML [4].  The MAGE Object Model describes the structure of the experiment, its components and relations. It is complemented by the use of standard collection of terms or ontologies.  The main ontologies used with MAGE are the NCBI Taxonomy [5], Gene Ontology [6] and the MGED Ontology [7].

At the moment of this writing the definition of a standard representation for microarray experiments is still undergoing significant development and new, more general, models and ontologies are being proposed (i.e.: the FuGE and FuGO projects)

### Microarray public repositories

Public repositories are intended to provide a persistent access to gene expression data produced by the scientific community. They are designed to collect data relative to heterogeneous experiments, hence the importance of the use of a proper annotation.  To enforce their role of knowledge repositories, major scientific journals requires that data supporting publications must be deposited on a public repository.

Array Express [8] has has been developed by the European Bioinformatics Institute (EBI) and has been modelled following the MAGE-OM model. It is a reference resource for the development of annotations.  Gene Expression Omnibus [9,10] has been developed by the US National Center for Biotechnology Information (NCBI). It stores high-throughput molecular abundance data (coming not only from microarrays).  CIBEX [11] is another repository from the Center for Information Biology and Data Bank of Japan. It is a MIAME compliant public repository, which stores a wide range of data, including mRNA based microarray data, gene expression data obtained with SAGE technology, and mass spectrometry proteomic data.

Public repositories are centralized resources that offer a public access to the community. They are not designed to support the needs of data management of single research groups.

### Microarray software

Many microarray database systems are available to the scientific community and are suited to be operated by small research groups. They vary in the features offered and in their characteristics. Almost all of them support MIAME experiment annotations, some present data analysis features and some include support for related laboratory activities (they are often included in the category of Laboratory Information Management Systems or LIMS).

Two widely adopted database systems are BASE [12] and maxD [13].  BASE is a microarry database system implemented as a web application. It offers LIMS functionalities and a set of data normalization and analysis features that can be extended thanks to a plug-in architecture. An optional module allows the management of custom built arrays. One limit of BASE is that it has a limited support for a particular class of microarrays (single channel) that include the Affymetrix GeneChip^® ^platform.  MaxD is composed by a set of tools that support different microarray data related tasks, such as data curation, data browsing and analysis.  MaxD supports a rich experiment description that can be customized in a particular installation by a responsible user (administrator). One limitation of maxD is its week support for a more complex scenario where multiple user groups have different access rights to the data.  Among many other microarray database solutions we cite Gecko [14], MicroGen [15], PlasmoDB [16].

Finally, several software tools exists that are specifically designed for the analysis of microarray data. These accepts as input tab-delimited text files, MAGE-ML files and sometimes they offer direct connection to specific databases.  The tab-delimited text file is the most common format. In this case all the experiment annotation is assumed to elaborated by the user before a selected set of data is analysed.  Among these tools we cite GeneSpring^® ^for its interactive graphical user interface, and Bioconductor, an entire collection of open source tools and libraries for microarray data analysis implemented in the R statistical language.

### Dendritic cells transcriptomics and the Genopolis Database

Within the Genopolis Consortium, we have used our database system to store information on dendritic cells.

Dendritic cells are professional antigen presenting cells that are central to the induction and regulation of immunity. Many genomic studies have been performed to interpret how Dendritic cells respond to microbial and non-microbial inflammatory stimuli. In kinetic experiments, gene expression profiles of immature in vitro derived mouse or human DC have been compared with gene expression patterns of activated DC at different times after challenge with the activation stimulus [17]. The analysis of the entire kinetic data sets has revealed that DC undergo a profound reorganization of gene expression in the first few hours after activation and then they progress versus a new resting state that is clearly distinct from the original immature DC state [18,19]. Improvement in the understanding of the functional complexity of DC maturation have been reached by the use of microarray experiments. This global studies have demonstrated the complexity of DC maturation at a molecular level [20,21].

For these reasons we have chosen to populate our database with data collected from unstimulated DC (different Dendritic cells subsets) and DC that have been treated with live organisms and with their component in a time dependent manner. To investigate the effects of different stimuli on DC function, we have used the Affymetrix GeneChip^®^. We took advantage of the previously described mouse DC line, D1 [22]. D1 cells are a splenic, myeloid and growth factor-dependent DC line that can be maintained indefinitely in culture in the immature state. This cell line can be driven to full maturation using different stimuli. Moreover it is composed of highly homogeneous cells.

## Implementation

### The data model

**Figure 1:**
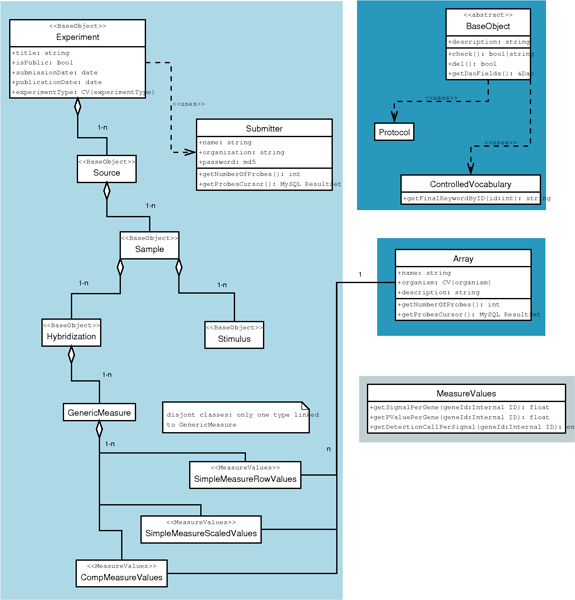
**The data model of the Genopolis Database**. The data model of the Genopolis Database. We represent data by means of a business objects layer. The representation is consistent with the MIAME recommendations and is similar to MAGE-OM. Respect to this two main simplifications have been introduced. First, we ignore chips manufacturing, since we make use of only one standard platform already described by Affymetrix. Second, we model the experiment design as a tree structure. Although this is not general (for instance, the same stimulus could be applied to two samples) it is a good compromise between flexibility and easy of use. We use controlled vocabulary and protocol classes to manage non free text fields.

The data model underlying the Genopolis Database maps a set of concepts in the experiment annotation to objects that are grouped according to a tree structure (Figure 1).

This arrangement is adequate for most experiment designs and single channels arrays. Its regular structure allows functions on the database content, such as consistency control, analysis and search to be implemented as simple functions on nodes that can be called in a tree traversal.

The objects implementing the experiment description are:

Submitter: the scientific responsible of an experiment.

Experiment: generic information about an experiment. Experiments are associated to Submitters.

Source: the biological source (organism, tissue, cell) under study. An Experiment can have one or more Sources.

Sample: a specific state of a source that is characterized by a time and a set of stimuli affecting this source at this time.

Stimulus: information regarding a stimulus applied to a source in an experiment. This includes the time of application of the stimulus and its duration. When the same stimulus affects more than one sample within an experiment, this object is repeated for each sample. This minor flaw was chosen in order to maintain the objects organized as a tree.

Hybridization: all information regarding the hybridization of a sample. This includes information on the array used (only the microarray GeneChip^® ^technology is supported) and the methods to extract and label the mRNA. At least one hybridization must be associated to a sample.

Measurement: a set of gene expression values derived from an hybridization. This includes information on the reading (scanning) of the microarray as well as the image analysis and normalization procedures used.

Other objects that are not organized as elements of a tree are used to define Protocols and Arrays.

Each element is characterized by several classes of attributes. Some attributes are simple named text or integer values, such as an animal identifier or an age value for a source. Some are relative to values that are defined in controlled vocabularies, such as the name of a cell line or of a tissue. Information on protocols and arrays used is defined in external objects that are referenced within the description elements. Finally each object accepts an informal natural language description to handle not explicitly supported information.

The Genopolis database object model is intended to describe experiments in terms of their building blocks. It then analyse the structure of its content to derive properties. For instance by default different hybridizations relative to the same sample are considered (and presented) as technical replicates, while distinct samples with the same stimuli and attributes (ex. time) are considered biological replicates.

### Architecture

The Genopolis database is realized as a relational database managed by a web based application.  The object model the database is based on is implemented by a set of software objects (business objects) that abstract the underlying relational tables. Hence, the resulting system is a n-tier architecture.  The current version of the Genopolis Database makes use of MySQL 4.1, but access to the SQL layer is standard and wrapped by the business objects, so that it would be easy to port it on different systems.  The core of the system is a web based application written in PHP4 and currently deployed on Apache and Linux based web servers.

In order to support the experiment annotation described later, two distinct relational databases are used.  One database stores incomplete experiment descriptions while these are being assembled. Another database contains data and descriptions of complete experiments and is available to the user for queries.  This distinction was made to improve reliability (provides a clean separation of data, even regarding unauthorized access and possible code flaws) and enhances performance, since read only instances of the database used for queries can be easily distributed on different machines, for instance on the nodes of a cluster.

The objects described above are organized in a tree structure and support recursive propagation of operations over the tree. One example of such operation is the checking of the consistency of the experiment description. This is implemented through an abstract *check() *method that is implemented for each object.  These objects also support rendering of information as HTML code for web forms (used for data submission) and for read only web pages. To implement this, each object representing an entity in the experiment description contains a list of objects corresponding to description items and implementing description types as strings, numbers, controlled vocabularies, free text, files. These objects are part of a distinct library called *daolib *(Data Access Objects), that allows the specification of their behaviour (i.e. Accepted values) and appearance (i.e. HTML rendering).

This Software Engineering based approach eases the maintainability and upgrading of the system.  The system maintains CEL files, image files and other attachments in a proper directory, and makes them available for download to authorized users. Measurement files are kept as files while assembling the experiment description, then parsed and stored in a single indexed MySQL table to support queries related to expression values.

Finally, other maintenance functionalities are implemented outside a client-server paradigm. These include import of GeneChip^® ^descriptions from Affymetrix MAGE-ML files (implemented in Java), transfer of data between the two databases, export of its content to ArrayExpress.

### Access control

**Figure 2:**
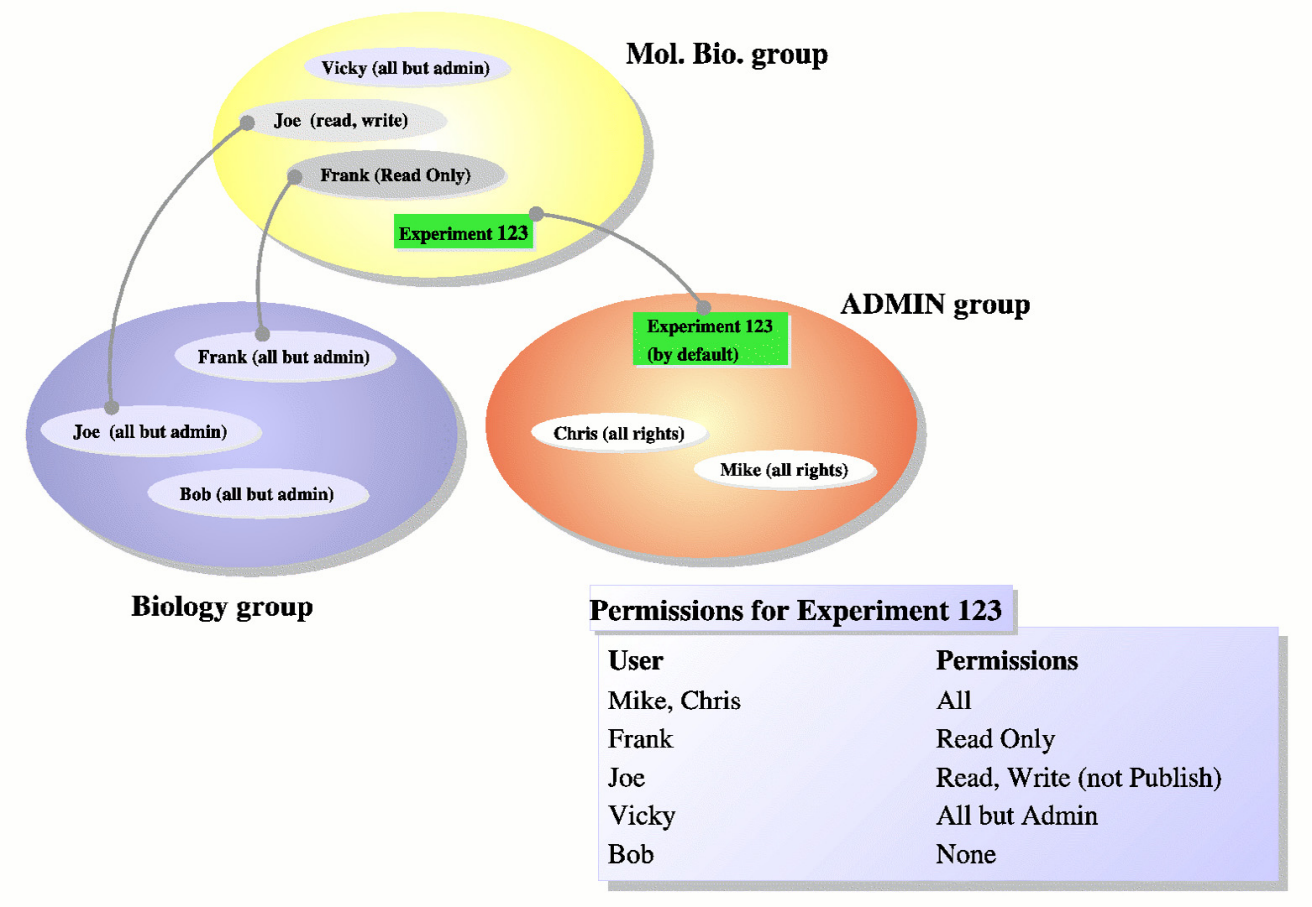
**Access management in the Genopolis Database**. Users belong to groups and each users/groups association has a related list of permissions. Experiments belong to groups. Access rights of a user to an experiment are determined by the combination of user membership and experiment membership.

The Genopolis database supports a flexible access schema to its content where users can be distinguished by group memberships and roles (Figure 2).  For instance, a data set may be declared accessible to the members of a given research group, and only accessible with limited rights (ex.: read only rights) to others.  In its current implementation the granularity of the access specification is the experiment: all annotation and data relative to elements that are part of the same experiment tree can be assigned as a whole to groups and users' access rights depend on their role within the group (administrator, protocol editor...).  This serves also as a support for a distributed annotation process: within a group, some users can be designated as responsible of the definition of protocols, controlled vocabularies, array annotations, while other users may be responsible for the experiment annotation.

The access system is based on a custom designed object oriented API. This is based on three PHP classes: GroupSecurityMgr (manages user groups), UserSecurityMgr (manages users and their association to groups, permissions associated to roles are defined here), ObjectSecurityMgr (manages experiments membership to the user groups).  API abstraction and customization classes (SecurityMgr, LoginManager) provide an easy to use access point to the programmer.

### MAGE-ML and ArrayExpress export

The Genopolis database can export its content in MAGE-ML. This feature has been implemented in order to provide an automated export to the ArrayExpess public repository.  The implementation of this functionality is based on Tab2MAGE. This tool, developed by the EBI, accepts the description of a single experiment in a simple tabular format and translates it into the equivalent MAGE-ML file.  Producing the structure of this kind of tabular files has been straightforward, since our experiment model is similar the model represented in them.  The support for controlled vocabularies has made possible their mapping to terms of ontologies accepted by ArrayExpress, such as the MGED Ontology. Integration of these ontologies within our system is undergoing.

### Deployment

The Genopolis database is currently deployed on a cluster architecture.  This is based on the Debian Linux distribution completed with the Web server load balancing software "Linux Virtual Server" and the high availability tool "Heart Beat".

Web users requests are transparently distributed to available service nodes. This distributes the web server load and ensures availability of the system even in case of nodes failure. Each node has a local copy of the database holding complete experiment description and data (these copies are read-only and updated when a new complete experiment description is added). This assures distribution of loads to different SQL engines and an optimization of data access.

## Results and discussion

We present here the features provided by the Genopolis Database and discuss how they support the implementation of a community database.

### Experiment annotation process

The Genopolis Database supports a community building a common knowledge base, by implementing a work-flow for data and experiment annotation, where different users can add different contribution depending on their role and responsibilities. Furthermore, it provides functions to check the consistency of its content and to dynamically create controlled vocabularies.  In detail, users with proper privileges can access a space where they can assemble experiments description and upload generated data. This can be done at different times, thanks to the ability of the system to save incomplete descriptions.  At any time users can ask the system to verify the completeness of the experiment description. Upon this request, the application verifies that all required information is present, that all the descriptions that need to be defined with terms from controlled vocabularies are fulfilling this condition, and it furthermore checks the content of data files for trivial errors (such as corrupted files).  It also verifies that some constraints are met (for instance, each sample must have at least one hybridization).  At the end of this verification process a report is generated and sent to responsible users.

When an experiment description is correct and all its data are present, a user can ask the system to make it available to the community (membership of users and their experiments to the community, as well as roles, are defined by a supervisor with proper privileges and responsibilities). In this case the entire experiment description is scheduled to be transferred to the complete experiments database, its measurements files are parsed and the copies on the cluster nodes are updated (this is done during low load times and it is automatically done by the MySQL replica service).

Some users within groups are responsible for protocols description, and a supervisor user is responsible for the curation of controlled vocabularies: new terms suggested by users in their experiment description are presented to this supervisor for approval. The supervisor can approve, deny or suggest new terms (note that this may be an iterative process in which the supervisor propose terms to be adopted by users).

### Data access and exploration

**Figure 3:**
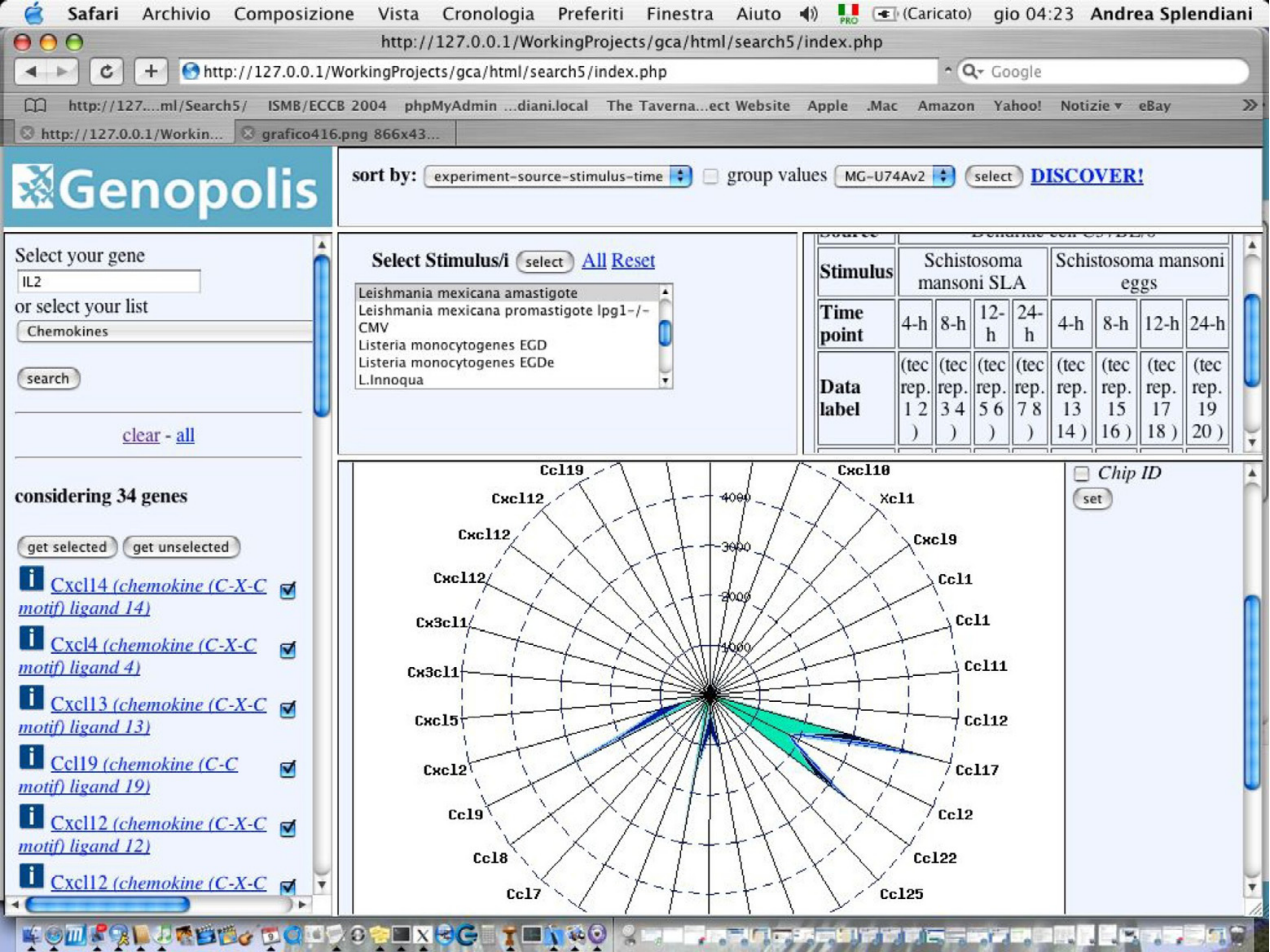
**Screenshot from the Genopolis Database visual query interface**. An example of a radar plot from the Genopolis Database visual query interface.

Several data access methods are provided by the database. One common idea in their design was to support intuitive and collaborative analysis of the database content. At any moment part of the database content can be exported as a configurable tabular file and imported in more sophisticated analysis tools.  An intuitive visualization interface provides a rich interactive access to the database content. Its basic idea is that gene expression can be studied analysing the association between set of genes and set of conditions [Figure 3].  The interface allows the user to browse interactively the data, to visualize expression relative to a given set of genes and conditions, and to "move" to other genes or conditions related by the expression data or by their annotation.

This interface resembles a microarray data matrix: a left panel presents a list of genes and allows their selection (genes may be searched by keywords, or selecting gene sets from predefined lists), an upper panel presents a list of samples and options for their selection and sorting, and a centre panel shows actual microarray values. This panel offers several visualization options that varies depending on the cardinalities of the set of genes and samples. For n samples and m genes it presents views as heatmap, radar plots, tabular files and lines, while if an element has a cardinality of two it presents also a scatter plot. When a huge number of genes is selected, as is the case for all the data relative to some conditions, only the tabular visualization is provided.

Both the genes lists and the samples list presents hyperlinks to information stored in the database (this is the case for instance of experiment description elements) and to external resources, such as NetAffx [23].

Many charts are provided with hyperlinks that popup information on the gene and condition relative to a single value. From this it is possible to navigate to related sets of genes. For instance, selecting a value for a gene under a condition will pop up a panel listing all the lists of genes (usually associated to functional groupings) this gene belongs to. It is then possible to change the current selection of genes in the left panel to one of these lists and to update all the information provided accordingly.

A "discover" function allows users to search for genes or samples with a similar expression pattern as for a relevant subset of the data matrix (this can be selected from the user). Genes or samples lists can be updated with the results of these queries and accordingly all the information presented is re-organized.

Overall, the Genopolis database provides tools where, starting from a set of genes and stimuli of interest, the user can browse the database content investigating interesting associations between genes and samples revealed by their expression values.

### Management of searches and data sets

Another interface provided by the Genopolis Database to access its data is the "Batch Query" interface. Here both genes and samples can be searched. The difference with the interface presented before is that this aims at providing finer search features, at the expenses of interactivity. It also aims at management of search results.

Concerning genes, sequence annotations can be queried using the usual SRS-like approach (based on Affymetrix annotations). Similarly, experiment annotation may be searched by keyword and relevant attributes.

In order to improve data management and collaboration, search results may be saved and later retrieved (it is possible to associate a search and a description to each result). Support for storing and reloading of predefined genes lists, such as genes functional families, is also provided, as well as the ability to operate on lists with intersection and union operators.  Saved search results are controlled by the access policy system, so that it is possible to define which user groups may have access (read-only or read-write) to them. Saved genes lists may be used in all the query interfaces by authorized users.

We have used this feature in our instance at the Genopolis Consortium to manage functional families of genes that are relevant to Immunology. This feature forms the beginning of a knowledge management system related to microarray data: for example, this makes possible for a researcher to share with his or her collaborators a list of genes he has found interesting while analysing some gene expression experiments. The batch query system has been implemented as a plug-in architecture that separates the code which search data, from the code which manages a search result. This makes easy to extend this interface and write new search functions or new data visualizations and operations.

### Export to public repositories

The Genopolis Database is designed as a community database and is intended to support group of users that trust each other and can share non public data. This is not in contrast and complements the role of public repositories.  In fact, we imagine our database being used to store a valuable collection of highly homogeneous data that can be shared (and analysed as whole) with confidence within a restricted community.  Once an experiment has been investigated and research results need to be published and disseminated, it can be automatically uploaded to ArrayExpress.

## Conclusion

The Genopolis Database is a valuable resource to assist a community in building a knowledge base of gene expression data and to support its analysis.  We have used it to implement a resource managed by the Genopolis Consortium to provide immunology relevant data to the scientific community studying dendritic cells. This provides a homogeneous data set with a coherent experiment characterization.

One relevant feature of the Genopolis Database is the ability to export its content to ArrayExpress (via a MAGE-ML export). This complements the vision of a community database in that it allows private data to be shared among trusted participants, and then published to a public repository as this data becomes publicly available.

We believe that the idea presented by our database system and its implementation can be a starting point for similar developments in other communities.

## Availability and requirements

At the time of writing access to the Genopolis Database is subordinate to a proper agreement and the code is available on request from the author.  We plan to open part of the database content to the public, and to make the software available on bioinformatics.org.

## Authors' contributions

AS has initiated this project and he is responsible for the overall design, the implementation of the core functions and the visual query interfaces.  MB is responsible for the batch query interfaces, the implementation of the access management schema, the ArrayExpress export tool (with contributions from AS), the Affymetrix MAGE-ML import and the daolib library.  GE is responsible for cluster adaptation and he is currently in charge of features development and users support.  MF and MM are responsible for the content and the operation of the running implementation of the Genopolis Microarray Database.  MP OB, NP and GM have contributed with ideas and feedback on the database design and implementation, as well as with their expertise in microarray data analysis.  FG and PC are responsible for the overall direction of the project and for its scientifc focus as a resource for immonology.
